# Animal Models, Therapeutics, and Vaccine Approaches to Emerging and Re-Emerging Flaviviruses

**DOI:** 10.3390/v17010001

**Published:** 2024-12-24

**Authors:** Thomas J. Baric, Z. Beau Reneer

**Affiliations:** Department of Epidemiology, Gillings School of Global Public Health, The University of North Carolina at Chapel Hill, Chapel Hill, NC 27599-3500, USA; tjbaric@email.unc.edu

**Keywords:** animal models, dengue virus, flavivirus, Powassan virus, tick-borne encephalitis virus, Usutu virus, West Nile virus, yellow fever virus, Zika virus, therapeutics, vaccines

## Abstract

Flaviviruses are arthropod-borne viruses primarily transmitted through the mosquito *Aedes aegypti* or *Culex* genus of mosquitos. These viruses are predominantly found in tropical and subtropical regions of the world with their geographical spread predicted to increase as global temperatures continue to rise. These viruses cause a variety of diseases in humans with the most prevalent being caused by dengue, resulting in hemorrhagic fever and associated sequala. Current approaches for therapeutic control of flavivirus infections are limited, and despite recent advances, there are no approved drugs. Vaccines, available for a few circulating flaviviruses, still have limited potential for controlling contemporary and future outbreaks. Mouse models provide us with a valuable tool to test the effectiveness of drugs and vaccines, yet for many flaviviruses, well-established mouse models are lacking. In this review, we highlight the current state of flavivirus vaccines and therapeutics, as well as our current understanding of mouse models for various flaviviruses.

## 1. Introduction

**Figure 1:**
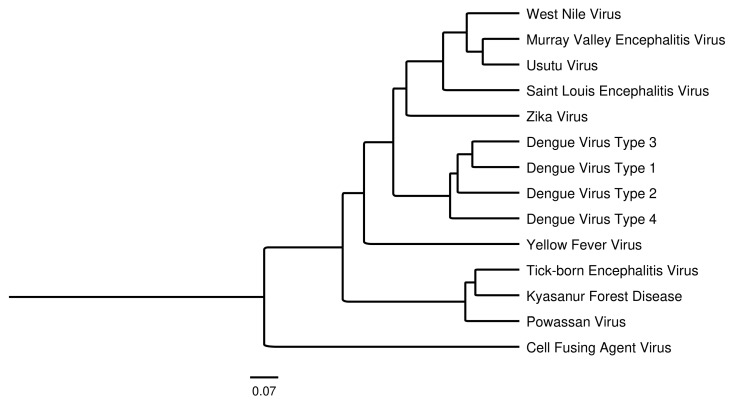
Phylogenetic tree of various flavivirus species depicting their genetic distance. Tree was created using MUSCLE alignment of full genome sequences in Genious Prime.

Flaviviruses are positive-sense single-stranded RNA viruses that are primarily transmitted through arthropod vectors. Among the more than 50 virus strains identified globally, dengue virus (DENV), Zika virus (ZIKV), West Nile virus (WNV), Japanese encephalitis virus (JEV), tick-borne encephalitis virus (TBEV), and yellow fever virus (YFV) are major human flavivirus pathogens. A phylogenetic tree based on full-length nucleotide sequences of these viruses, as well as additional species, is shown in Figure 1. Of these flaviviruses, dengue is distributed across the temperate regions of the globe where it currently imposes a significant disease burden with estimated case numbers >390 million globally/year and cases are predicted to continue increasing [1,2]. Dengue by far has the most cases when compared to other notable flaviviruses (Table 1). As many as 4 billion people may be at risk of contracting dengue, especially as global temperatures continue to rise. In the US, global warming has extended the mosquito season about 10–17 days between 1980 and 2023, especially along the East Coast, Northern, and Pacific states [3]. The *Aedes* genus mosquitoes are the principal vectors that mediate transmission of dengue, Zika, yellow fever, and other flaviviruses to humans, and their broad geographical distribution places much of the human population at risk globally in tropical and subtropical regions [4]. Although less prevalent, some flaviviruses can be transmitted through tick bites, such as tick-borne encephalitis and Powassan virus (POWV). Flavivirus infections produce several unique diseases, which can result in asymptomatic to life-threatening illness depending upon the virus, strain, and host susceptibility. Disease can manifest itself in several different ways. The majority of dengue cases are uncomplicated and often go undiagnosed, but a small portion of individuals develop more severe disease usually identified by plasma leakage. Dengue infections are classified into three categories, undifferentiated fever, dengue fever (DF), and dengue hemorrhagic fever (DHF). DHF is then further classified into four levels of severity, with grades III and IV including dengue shock syndrome (DSS). Dengue fever often presents a high fever, along with joint pain and rash, while severe cases are often classified by shock, respiratory distress, severe bleeding, and impaired consciousness and organ function [5]. Zika infections usually present as mild or asymptomatic cases, but during pregnancy can cause congenital birth defects such as microcephaly, still births, and miscarriages; rarely, it can also cause Guillain–Barré syndrome [6]. West Nile virus cases are normally asymptomatic but sometimes show fever, fatigue, muscle pain, and gastrointestinal complaints. In severe cases, WNV can also cause neuroinvasive diseases such as meningitis and encephalitis [7]. Japanese encephalitis virus disease symptoms are majorly asymptomatic or nonspecific. Early cases of encephalitis exhibit coryza, diarrhea, and rigors, then followed by the more severe symptoms such as encephalitis, Parkinson-like features and other movement disorders, and seizures [8]. Tick-borne encephalitis virus tends to be asymptomatic or nonspecific as well, with severe clinical manifestations showing flu-like symptoms and reoccurring fever and signs of CNS involvement lasting up to 21 days. Eventually, the disease progresses to meningitis, meningoencephalitis, inflammation, and pain along the spinal cord [9]. Some flaviviruses also showcase a phenomenon where preexisting antibodies to a closely related strain can enhance disease severity, caused by an antibody-dependent enhancement mechanism (ADE) [10]. The frequency of ADE is unclear, but estimated to occur in ~1–4% of secondary DENV infections, primarily driven by low antibody concentrations that bind but not neutralize, and then promote virus binding of Fc receptors on immune cells, leading to infection [11].

The collective economic burden of these distinct flaviviruses varies and is difficult to estimate due to the disparity in data from different countries. Dengue’s global economic burden, which primarily stems from the loss in productivity, is estimated to be USD 8.9 billion per year, with other estimates approaching USD 40 billion [12,13].

**Table 1:** A list of notable flaviviruses that cause high morbidity in endemic populations.

Global Statistics					
Virus	Cases	Severe Infection Chance ^7^	Fatality Rate	Vector	Source
Dengue Virus	100–400 m	<5%	1.10%	*Aedes aegypti*	[14,15,16]
Zika Virus	<1000	5–14% ^1^	10% ^2^	*Aedes aegypti*	[17,18]
West Nile Virus	-	<1%	4–14%	*Culex* spp.	[19,20]
Japanese Encephalitis Virus	30–50 k	<1%	20–30%	*Culex tritaeniorhynchus*	[21,22]
Tick-borne Encephalitis Virus	10–12 k	2–30%	2 ^3^, 6–8% ^4^	*I. ricinus* ^3^/*I. persulcatus* ^4^	[23,24,25]
Yellow Fever Virus	84–170 k ^5^	10–15% ^6^	20–50% ^6^	*Aedes aegypti*	[26,27]

^1^ Risk of congenital Zika syndrome in newborns. ^2^ Fatality rate reported in children with microcephaly/congenital Zika syndrome. ^3^ European subtype. ^4^ Siberian subtype. ^5^ Number of severe cases reported. ^6^ Rates reported for total number of cases. ^7^ Severe infection is defined as needing hospital care.

The absence of safe, effective vaccines for the majority of, but not all, flaviviruses leaves the primary method for disease mitigation to public health intervention strategies like vector control and supportive clinical care. Mouse models have proven reliable over decades’ worth of research and provide critical insights into the pathogenesis of flaviviruses. Mice are a cheap and easily obtainable way to recapitulate disease in a living organism. The treatment status for these viruses varies; yellow fever, Japanese encephalitis, and tick-borne encephalitis all have effective vaccines. Dengue virus vaccines, like Dengvaxia and QDENGA^®^ (TAK-003), have been approved for use in some countries but concerns about ADE disease enhancement in naïve populations remain high [28]. In contrast, Zika and West Nile viruses lack human vaccines, although animal vaccines like PRESTIGE^®^ WNV (for horses) offer hope that one of the human WNV vaccines (e.g., ChimeriVax-WN02, inactivated; NCT00442169) or ZIKV vaccines (e.g., ChinZIKV, mRNA-1325; NCT03014089, and mRNA-1893; NCT04064905) that are currently in phase 2 and phase 1 clinical human trials, respectively, will prove successful.

## 2. Flavivirus Characteristics

### 2.1. Virus Structure

**Figure 2:**
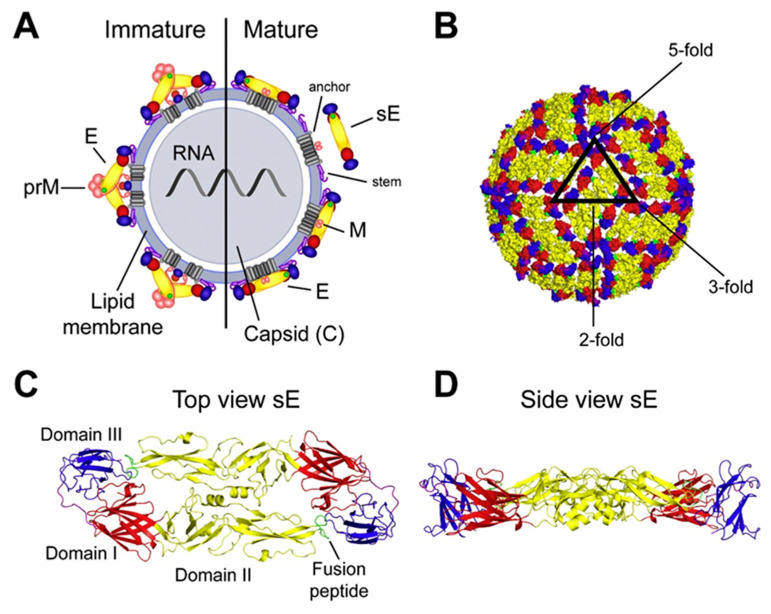
Diagram of flavivirus structure. (**A**) Cross section of flavivirus virion showing organization of three structural genes: Envelope (E), Premembrane, Membrane, and Capsid (C). The left side represents the immature virion, and the right side represents the mature virion. (**B**) Structure represents the symmetry in organization of the envelope dimers. (**C**) Ribbon diagram of envelope dimer with envelope domain I (EDI) in red, envelope domain 2 (EDII) in yellow, and envelope domain III (EDIII) in blue. (**D**) Side view of the envelope dimer [33].

Flavivirus particles take on spherical or pleomorphic virion morphology with an internal icosahedral shape, surrounded by a lipid envelope encoding viral spike glycoproteins. Their genome is ~11 kb in length and encodes a single large open reading frame. The genome encodes three structural genes, encoding the capsid (C) protein, and the pre-membrane (prM) and envelope (E) glycoproteins (Figure 2A). In addition, seven non-structural proteins (NS1, NS2a, NS2b, NS3, NS4a, NS4b, NS5) are encoded within the genome. Flaviviruses like DENV can take on two primary structural conformities: immature and mature (Figure 2A). The immature structure is not smooth like its mature counterpart and can be found both within and outside of cells [29]. Immature flavivirus particles have 60 irregular trimeric spikes crafted from prM/E [30]. These immature particles are non-infectious intermediates and are unable to fuse to the host membrane during entry [30]. However, fully immature particles can become infectious when complexed with anti-prM antibodies, providing a potential mechanism for escape from neutralizing antibodies in preimmune individuals. Underneath the outer layers of the envelope and membrane proteins lies the nucleocapsid. The nucleocapsid consists of a single copy of the positive-sense RNA bound to multiple copies of the capsid protein [31,32].

Mature flavivirus virions form when prM is cleaved into pr and M and are organized into a smooth external shell composed of multiple glycosylated E protein monomers that self-assemble into E dimers and trimers, and which encode highly conserved structural domains [34]. Each monomer encodes three distinct structural domains referred to as EDI, EDII, and EDIII (Figure 2C) [35]. The monomers self-assemble to form anti-parallel homodimers; three adjacent homodimers create a “raft” on the surface of the virion. Importantly, each of the domains on the E protein contain epitopes for neutralizing antibodies. In some flavivirus strains, EDIII has been shown to encode epitopes that elicit potent neutralizing antibodies, while in other strains potent neutralizing and cross-neutralizing antibodies target EDI or EDII [36,37].

### 2.2. Nonstructural Proteins

The nonstructural protein 1 (NS1) protein plays a significant role in the flavivirus lifecycle. In replication, NS1 is likely involved in the replication complex because it has been shown to co-localize with dsRNA in replication vesicles [38]. NS1 has also been associated with NS4A to facilitate viral replication and fitness [39,40]. A unique feature of several flavivirus NS1 proteins, like DENV, is that secreted forms cause vascular leakage through the disruption of the endothelial glycocalyx-like layer (EGL) [41]. During vascular leakage, NS1 is endocytosed into a cell in a dynamin- and clathrin-dependent manner [42]. Secretion of the NS1 protein into the extracellular matrix attenuates both the classical and lectin pathways by interacting with the complement system to degrade anaphylatoxin C4 into C4b, protecting dengue from complement-dependent neutralization [43]. Importantly, NS1 is highly conserved and anti-NS1 antibodies can protect animals against flavivirus infection, so it has become a target of interest for therapeutic treatments and vaccine designs, further discussed below [44].

Flavivirus NS2 codes for NS2a and NS2b. NS2 is a host-regulated autoprotease that cleaves NS2-NS3. NS2a and NS2b function in RNA synthesis and virion assembly. Both proteins can colocalize with dsRNA, which suggests they are involved with the viral replication complex. Importantly, NS3/NS4A complexes or uncleaved NS2-3 in complex with NS4A (NS2-3/4A) represent essential components that facilitate the switch between RNA replication and virion formation [45].

The NS3 protein antagonizes interferon responses and possesses an N-terminal serine protease and a C-terminal helicase domain; the latter activity unwinds double-stranded viral RNAs. Both are highly conserved among flaviviruses [46]. NS3–NS2b interactions are vital for polyprotein processing and RNA replication. When using NS2B as a cofactor, NS3 proteolytically processes the large genome length flavivirus polyprotein into individual structural and nonstructural proteins, with some assistance from other cellular proteases [47]. The first-in-class inhibitor, JNJ-1802, blocks DENV NS3–NS4B interactions within the viral replication complex, inhibiting virus replication and growth in vitro and in vivo [48]. JNJ-1802 is currently in phase 2a human clinical studies and showing promise. NS5 antagonizes interferon signaling and encodes methyltransferase and RNA-dependent RNA polymerase activities that are essential for genome replication [49,50].

The NS4A and NS4B proteins have been shown to be a determining factor in the rearranging of cytoplasmic membranes, notably NS4A being responsible for the formation of both convoluted membranes and vesicular packets [51]. The NS4A protein plays a major role in virus replication and is required for formation of the replication complex [52]. NS4A and NS4B both play a role in inhibiting the interferon response and can enhance replication when expressed alongside IFN-sensitive viruses [53]. In WNV, NS4A and NS4B also stimulate the unfolded protein response, leading to an increase in ER volume, chaperone proteins, and protein degradation, all of which enhance viral replication [54,55].

### 2.3. Flavivirus Receptors

The first step in flaviviral entry to a cell is the recognition of the appropriate cell receptor(s), including αvβ3 integrins and many others, and are subjects of multiple reviews [56,57,58]. This wide range of flavivirus receptors engages the envelope (E) protein, an adhesive molecule located on the envelope of the virion [59]. One well-studied receptor that flaviviruses recognize is heparan sulfate. It is a sulfated polysaccharide known to be a common receptor among multiple flaviviruses and plays a role in cell entry by congregating viral particles on the cell surface [60,61], although some viruses can acquire heparan sulfate binding after serial passage in cell culture [62]. Insect cells tend to be more restricted in their receptors; a few examples are 48kDa tubulin-like proteins and 70kDa heat shock proteins found on the surface of insect cells [59,63,64]. Human receptors include the human dendritic cell receptor ICAM3-grabbing non-integrin (DC-SIGN), TIM-1 and TAM, and AXL receptors [61,65,66,67]. DENV receptors include carbohydrates, heparan sulfate, integrins, claudin-1 cell receptor, and C-type lectin receptors like DC-SIGN/L-SIGN, depending on the cell [59]. Alternatively, multiple receptors may contribute to entry in a systematic process involving initial weak interactions that facilitate subsequent engagement networks that engage one or more additional entry receptors. Other flaviviruses such as WNV, JEV, DENV, and ZIKV can also gain cell entry by antibody-dependent enhancement through the recognition of Fc receptors which are found on the surface of various immune cells [68].

### 2.4. Host Innate Response

Infection of flavivirus triggers the host innate immune response, leading to cytokine production which includes interferons (IFNs) which regulate and activate the host innate and immune response to produce an antiviral state that interferes with viral replication [69]. Activation of IFNs relies on a group of proteins known as pattern-recognition receptors (PRRs) that are expressed on external and internal membranes and in the host cytoplasm and recognize structures that are conserved among microbial and viral pathogens called pathogen-associated molecular patterns (PAMPs) [70]. IFNs utilize the JAK/STAT pathway for signal transduction and can be grouped into three types (I, II, III), which differ based on their receptor complexes [70]. When IFNs bind to the surface of a cell, they trigger the transcription of hundreds of interferon-stimulated genes (ISGs) that inhibit virus infection, stimulates adaptive immune responses, and initiates reparative processes in diseased tissues [50,70]. Flaviviruses encode protein functions that antagonize and evade type-I IFN signaling. Strategies include an NS5 methyltransferase activity that introduces a 2′-O methylated 5′ cap on the flavivirus genome length RNA. This 5’ cap modification functions to mimic host RNA, thus avoiding detection by interferon-induced tetratricopeptide repeat (IFIT) proteins [71,72]. Nonstructural proteins also play a part in flavivirus immune avoidance. The NS4B protein, encoded in DENV, WNV, and YF, can partially block IFN-β signaling by inhibiting phosphorylation of STAT1 [73]. Additionally, in WNV, NS4B has been shown to stimulate the unfolded protein response (UPR) in cells, which consequently inhibits type-I IFN signaling, as well as inhibiting JAK/STAT signaling [54,74]. Other flaviviruses encode other novel strategies to evade host innate immunity, discussed in more detail in earlier reviews [69,75,76].

### 2.5. Animal Models

Robust small animal models that recapitulate human disease phenotypes are critical for vaccine and countermeasure development. Mice provide valuable insight into immunological and genetic factors that dictate disease progression. While there are obvious differences between both mice and humans, if they are acknowledged, mice can be used to predict how disease develops or the effect of treatment on humans [77]. For this reason, mice play a vital role during the FDA approval process during preclinical research to test the effectiveness of novel medications before proceeding into human trials. A summary of mechanisms studied in various mouse models has been provided (Table 2). Many of these animal models provided the necessary means to evaluate vaccines and therapeutics mentioned in the next section. Flaviviruses replicate with varying degrees of efficiency in mice with some strains causing life-threatening disease in standard laboratory strains, while other strains require mouse adaptation, oftentimes in combination with defects in host immune signaling. WNV has been heavily studied in various murine laboratory strains, which have shown to be an effective tool for modeling infection [78,79,80]. Mice infected with WNV develop a systemic infection initially thought to have begun as replication in the Langerhans dendritic cells following an intradermal inoculation [81]. The virus eventually enters the blood stream and spreads to the gut, spleen, kidneys, and surrounding tissues, eventually entering the central nervous system (CNS). Previous research has shown that C57BL/6 (B6) and BALB/C mice can accurately mimic severe and CNS infection phenotypes that develop in humans [82,83,84]. Mice are also able to accurately recapitulate host susceptibility and age-related risk factors involving WNV infections in humans [85,86]. Although these mouse strains have proven useful for understanding WNV pathology, they are limited in their ability to represent the full breadth of the disease [80,87,88,89]. Importantly, the collaborative cross (CC) is a genetic reference population derived from eight founder strains and the resource is specifically designed to identify complex genetic traits [90,91]. An advantage to using CC mice over traditional mice models is that the genetic background of these mice varies and allows researchers to uncover roles of genetic factors in disease phenotypes [92]. CC (032x013) F1 recombinant inbred intercross (CC-RIX) mice develop a variety of unique acute and persistent disease state. Unlike some other mouse strains, the CC (032x013) mice can more accurately reflect the diversity of infections in outbred human populations [80,92,93,94]. These studies showed that altered innate immune responses and increased frequencies of regulatory T cell subsets, and lower expression of genes associated with cytolysis, were correlated with the development of chronic WNV disease [80].

**Table 2:** Summary of mouse models and mechanisms of interest.

Virus	Animal Model	Mechanism Addressed	Source
Dengue Virus	BALB/c	Pathogenesis of tissues	[95]
Dengue Virus	BALB/c and C57BL/6	Age-related pathogenesis	[96]
Dengue Virus	AG129	Antibody-dependent enhancement	[97]
Zika Virus	C57BL/6	Neonatal pathogenesis	[98]
Zika Virus	C57BL/6 IFNAR^-/-^	IFN-α/β signaling	[99]
Zika Virus	C57BL/6 IFNAR^-/-^	Insect to mouse transmission	[100]
Zika Virus	Anti-IFNR1-treated Rag1^-/-^	Vertical and sexual transmission	[101]
Zika Virus	CC Mice	Genetic factors in pathology	[102]
West Nile	Gold Hamster	Encephalitis	[78]
West Nile	Outbred Swiss mice [Arc(S)]	Pathogenicity between strains	[79]
West Nile	CC RI	Chronic infection	[80]
West Nile	BALB/c	Pathogenicity and neuroinvasiveness	[84]
Tick-borne Encephalitis Virus	BALB/c/STS/CcS-11	Genetic factors in pathology	[103]
Tick-borne Encephalitis Virus	B6 IL-10KO/TNF-α KO	Cytokines	[104]
Tick-borne Encephalitis Virus	C57BL/6j	Systemic inflammatory and stress responses	[105]
Japanese Encephalitis Virus	C57BL/6	Pathogenesis	[106]
Japanese Encephalitis Virus	Not Stated	Encephalitis and disease progression	[107]
Japanese Encephalitis Virus	BALB/c	Contract transmission	[108]
Yellow Fever	C57BL/6 PVR-Tg21 IFNAR^-/-^	Immunopathogenesis	[109]
Yellow Fever	A129	Viscerotropic infection	[110]
Yellow Fever	AG129	Neurotropic disease	[111]
Yellow Fever	AG129	Neurotropic and viscerotropic disease	[112]
Yellow Fever	CD-1	Fetal development	[113]
Yellow Fever	hSTAT2 KI	Immunocompetency	[114]

ZIKV infection into wild-type mice is typically unsuccessful due to ZIKV’s inability to degrade mouse STAT2, allowing the activation of downstream antiviral genes to prevent efficient replication [115]. However, mouse-adapted strains of ZIKV have been developed and ZIKV infection into neonatal wild-type mice is possible and could be advantageous when studying neurological effects during brain maturation due to brain development that occurs outside of the womb [116]. ZIKV infection into neonatal immunocompetent C57BL/6 mice inoculated one day after birth resulted in non-fatal neurological disease that included kinetic tremors, ataxia, seizures, and unsteady gait, as well as neurodegeneration in the cerebellum at 13 dpi, which symptoms gradually diminished [98]. These results contrasted with infection in 5–6 week of age wild-type (WT) mice who showed no weight loss, morbidity, or mortality. Additionally, mice who were also lacking MAVs and IRF-3 showed similar results [99]. Immunocompromised mice with interferon receptor deficient (*Ifnar^-/-^*) gene have proven a useful model in studying ZIKV in adult mice [100,117]. Interestingly, 6-week-old C57BL/6 *Ifnar^-/-^* showed no difference in viral RNA levels between mosquito transmission and subcutaneously through footpad injection [100]. ZIKV can also affect human offspring and be sexually transmitted, which adds an additional two factors that need to be represented in mouse models [99,118]. Sexual transmission of ZIKV has been demonstrated between infected immunocompromised males to naïve immunocompromised females, resulting in infection of the female reproductive tissues [101]. Vertical transmission in immunocompromised mice has shown virus to be found in the uterus and placental tissue [101]. Fetuses were observed to have decreased head and body size; placental analysis found apoptosis of trophoblasts and endothelial cells, vascular leakage, and placental insufficiency [119]. Robust primate models of ZIKV in pregnant animals recapitulate many of the serious neurological birth defects seen in humans [120].

DENV-immunocompetent mouse models are resistant to DENV through similar mechanisms as ZIKV and are unable to sustain infection or symptoms but can still be useful [121]. Intravenous inoculation of DENV into BALB/c mice resulted in viral genome being found in the heart, skeletal muscle, and lung tissue with histological samples being comparable to human cases [95]. Subsequent infections with heterologous DENV strains in BALB/c mice can lead to heart damage [122]. Different immunocompetent mice strains exhibit variation in susceptibility to DENV [96]. Immunodeficient mice lacking interferon receptors I and II have proven useful models for ADE after infection with a mouse-adapted DV2 strain, D2S10 [97]. AG129 mice inoculated with DENV serum or poorly neutralizing antibodies resulted in enhanced infection; similar results were also achieved by incubating virus with antibodies before injection [97]. More robust primate models have been developed; however, the more serious clinical outcomes of DENV in humans remain limited, highlighting the need for improved animal models of disease [123,124].

Mouse models of TBEV have also proven useful tools to model disease phenotypes and natural genetic variation in resistant STS and susceptible CcS-11 mice regulate infection and disease severity [103]. Immunodeficient mice lacking tumor necrosis factor alpha (TNF-α) and interleukin 10 (IL-10) also developed more serve disease than wild-type mice [104,105]. In addition, CC mice display polygenic control of flavivirus susceptibility and pathogenesis as CC071 mice, but not other CC strains, show increased susceptibility to ZIKV, DENV, POWV, and WNV as well as Norway rat hepacivirus chronic infection [102,125,126].

While some mice are not susceptible to a natural JEV isolate, a mouse-adapted strain of JEV can be created through multiple passages in neonatal mice to allow infection in otherwise resistant 3–4-week-old C57BL/6 mice [106]. This JEV-S3 strain expresses viral antigens and RNA, and causes clinical symptoms and progressive cellular damage in the mouse brain [106]. Route of infection in mice can drastically impact outcomes with some leading to little if any clinical disease whereas footpad inoculation in 2- and 5-week-old mice showed more consistent disease progression [107,108]. JEV animal model development has resulted in highly variable outcomes and more research is needed into the development of these resources for countermeasure development [127].

YF infections are generally unsuccessful in immunocompetent mice regardless of inoculation route, likely reflecting poor NS5 antagonism of murine STAT2 signaling [109,128]. Therefore, immunodeficient mice such as AG129 mice which lack INF-α/β and INF-γ receptors have provided alternative models for the study of YF [110,111]. Limited evolution of YF has also been seen following serial passage in mice, allowing for minimized risk of resistance development against vaccines or antivirals [111]. Infection of 17D-YF results in uniform lethality with high viral titers in the brain and liver, and death within 6–7 days [110,112]. Interestingly, viral loads do not appear to correlate with disease severity in INF-α/β receptor knockout mice [109]. Mice have previously been used to study the effects of YF vaccination on pregnancy in mice, finding that vaccination had no adverse outcomes, except when administered during the early gestational stages of pregnancy [110,113]. Mouse-adapted flaviviruses have also provided for the development of efficient small animal models for ZIKV and DENV, although such models still require some attenuation of innate immune responses by introducing human STAT2 into mice (hSTAT2 KI) or the use of AGM129 mice [114,129].

## 3. Therapeutics

There are no approved therapeutics for flavivirus infections and only a small number of flaviviruses have licensed vaccines. In other cases, the development of new vaccines has been hampered because of limited market potential or from concerns centered around the potential of complex vaccine-induced immune complications [130]. Current treatment is only limited to clinical management of the complications that arise from infection. Dengue is challenging because the risk of vaccine-induced ADE may worsen disease in vaccinated individuals or individuals who have had a previous history of other flavivirus infections such as ZIKV, which has also been shown to enhance dengue in non-human primates [131,132]. Research is ongoing to determine if ADE is caused by other flaviviruses in humans. Administration of flavivirus drugs is complicated because of limitations in diagnostic tools, speed of results, unstable governments, and availability of tests, reducing capacity for flavivirus detection in resource-limited nations [133,134]. This highlights the need for effective therapeutics to be developed until a safe and effective vaccine can be designed.

### 3.1. Antiviral Drugs

There are no drugs made specifically for the use of treating flaviviral infections, although there have been many in vitro experiments carried out showcasing reduction in inhibition [135]. Research for anti-flavivirus drugs is either focused on targeting the virus-encoded or host proteins [136]. General antiviral strategies involve disruption of the disease pathway, either by inhibiting the virus replication or by targeting critical host proteins/networks that contribute to virus replication/pathogenesis, or by immune modulation. Direct-acting antivirals (DAAs) and host-targeting antivirals (HTAs) are the two classes of antivirals that encompass drug therapies for virus infections. A benefit of DAAs is the low potential toxicity to the host but viruses may rapidly evolve drug resistance mutations due to low fidelity rates of the viral RNA-dependent RNA polymerase [137]. Antiviral drugs function to prevent the formation or function of virus replication complexes, entry, or assembly or inhibit key enzymatic activities [138]. HTAs present the advantage of broad-spectrum antiviral activities for functions involved in virus assembly and are generally not susceptible to drug resistance like DAAs. While this makes HTAs useful for treating new emerging viruses, the inhibition of cell proteins, interactions, and signaling networks may elicit a variety of toxicity concerns and side effects [139]. The genetic variation in outbred human populations may also result in altered drug performance [140]. Flavivirus viral proteins most frequently targeted for antiviral design include E, NS1, NS2B, NS3, NS3-, NS3–4b interaction, and NS5, mainly targeting their critical functions in replication and pathogenesis. Inhibition of the structural E glycoprotein is one ideal target because of its role in viral entry, membrane fusion, and the release of RNA into the cytoplasm. Small molecules or monoclonal antibodies that interfere with E glycoprotein receptor interactions block infection, thereby blocking virus amplification and viremia, while avoiding downstream complications that arise when antiviral drugs function intracellularly. Drugs inhibiting the envelope protein has already been demonstrated as an effective treatment in other viruses, although rapid selection for drug attenuating mutations has reduced enthusiasm [141].

The replicase enzymatic machinery is often considered a premiere target for the development of robust small molecule inhibitors against flaviviruses. The NS5 protein is responsible for interferon suppression, genome replication, and RNA capping [142]. Drugs targeting the NS5 RdRp have already been demonstrated in other viruses such as SARS-CoV2 and HIV [143]. In particular, remdesivir has demonstrated potent in vitro activity against multiple coronaviruses, flaviviruses, picornaviruses, and filoviruses via delayed chain termination of nascent viral RNA, supporting the development and testing of other nucleoside inhibitors that block virus replication [144,145,146]. Alternatively, proteases NS2B and NS3 and/or their interaction partners (NS3-NS4b) also make appealing targets because they are highly conserved across the flavivirus genus and play a critical role in the cleavage of multiple viral proteins during replication. JNJ-1802, a first-in-class oral pan-DENV antiviral, blocks the NS3–NS4B interaction within the viral replication complex at low nM activity and prevents DENV replication and pathogenesis in mice and primates in vivo [48]. A phase 1 human trial supported further clinical development as a prophylactic and therapeutic DENV drug [147]. JNJ-1802 is currently in a phase 2a DENV3 human challenge study. The compound has also recently progressed into clinical trials at multiple sites in 10 countries, including the Philippines, Thailand, Peru, Brazil, and Colombia.

Ideally, small-molecule drugs would be stable, have a good shelf life, be taken orally and once daily, and have superior pharmacologic data. With respect to ZIKV infection, antiviral drugs should be safe to take before or during a pregnancy to prevent congenital zika syndrome and be harmless to the developing fetus. Ivermectin has been shown to be an effective general antiviral by targeting the host heterodimeric importin (IMP) α/β1 complex inducing conformational changes, inhibiting the binding of IMPα to IMPβ1 [148]. With minimal side effects, ivermectin has been shown to inhibit all DENV serotypes and other flaviviruses as well as attenuating replication through interactions with the NS3 and the NS5 protein [149,150]. Other molecules that target the IMPα/β1 pathway and have shown interactions with flaviviruses are GW5074, gossypol, 4-HPR, and mifepristone [151,152,153,154]. However, these studies are confined to cell culture-based assays, limiting enthusiasm. Moreover, the in vivo potency of this class of compounds requires additional scrutiny in rodents and primates before moving into human trials, especially since ivermectin also had potent in vitro antiviral activity against SARS-CoV2, yet showed no clinical benefit in well-designed human studies [155]. Rather, over 1700 adverse reactions in ivermectin-dosed COVID-19 patients were reported in the first two years of the pandemic, resulting in several deaths [156]. Consequently, caution is strongly warranted.

### 3.2. Monoclonal Antibodies

Human monoclonal antibodies (hmAbs) are produced from plasma blasts and memory B cells when an immune response is provoked after infection or vaccination. Passive transfer of these purified antibodies either prior to or soon after an acute viral infection attenuates virus pathogenesis and mortality. Not surprisingly, the Food and Drug Administration (FDA) has reviewed and approved products for the treatment of a variety of viral infections, including RSV and SARS-CoV2 [157]. Human monoclonal antibodies typically have robust neutralizing activity associated with the targeting of key epitope on the virus that prevent receptor binding, or virus fusion and entry, thereby limiting virus replicating and pathogenesis [158]. MAbs are typically administered by intravenous infusion and are highly specific, resulting in low toxicity in humans. They can also be produced quickly in large quantities that are sufficient for broad use across human populations [159,160]. Research into modified Fc receptor mAbs has also given the ability to extend the half-life of a treatment 2–4-fold, allowing an additional 100 days of circulation, potentially allowing the development of a longer lasting antibody to treat flaviviruses [161]. A primary advantage of mAb treatment is that it can be used to prevent infections, and therapeutically reverse disease symptoms and reduce virus spread, when other treatment options are not available.

MAbs treatment has been shown to be a potential treatment option against flavivirus infection in mice [162]. Nevertheless, weak or low neutralizing antibody concentrations may initiate ADE following secondary DENV infections. Early on, most antibody therapies against flaviviruses target EDIII of the envelope protein. EDIII antibodies are highly type-specific and have low cross-reactivity [163]. Importantly, potent quaternary neutralizing antibodies have been described that bind epitopes in EDI and EDII and also provide potent protection from infection in animal models of human disease [164,165]. Comprehensive analysis of the DENV3 virus E glycoprotein domains that are targeted by type-specific neutralizing antibodies identified an array of epitopes in EDI, EDII, and EDIII, including potent EDII antibodies that protect in vivo, demonstrating a need for more comprehensive analyses of the numbers and locations of neutralizing epitopes in other DENV serotypes. In addition to type-specific neutralizing antibodies, potent, broadly cross-neutralizing antibodies, like C10, exist that target envelope dimer epitopes that span multiple monomers in DENV serotypes, and ZIKV, thereby providing another option for therapeutic control [165,166]. Other mAbs that target other proteins, such as M and NS1, protect against lethal outcomes in mice and are also being considered as therapeutic candidates [163,167,168,169,170].

Using WNV as a model, E16 is an anti-WNV antibody that was isolated from mice that recognizes conserved region of 16 residues on EDIII of WNV. It is believed that E16 blocks the conformational changes in the E protein after binding and can protect against lethal outcomes in mice when administered late in infection [168]. WNV-86 is another potent WNV antibody that shows potential for therapeutic purposes. WNV-86 binds to E protein EDII, recognizes mature virions lacking prM, and protects against lethal infection outcomes in mice [171,172]. For ZIKV infections, the human monoclonal antibodies ZIKV-117 and ZKA64 bind to EDIII and EDII, respectively [167,173]. Treatment of ZIK-117 reduced tissue, placental, and fetal pathology in mice, and mortality. Treatment of ZKA64 prevented mortality in A129 mice when administered one day prior to or after infection. ZKA64 demonstrates low cross-reactivity with DENV viruses, necessitating the need for additional studies that access off-target ADE phenotypes. Antibodies binding to ZIKV NS1 protein are oftentimes Zika virus-specific and provide potential tools to diagnose productive infections [167]. In summary, therapeutic human monoclonal antibodies targeting flaviviruses represent a robust and portable approach to control infection in humans. The main limitation appears to be market potential and delivering drugs to regions of the world that are most prone to tick- and mosquito-borne flavivirus diseases.

## 4. Vaccine Approaches

The long history of human flavivirus epidemics, coupled with the likely emergence of new flaviviruses into naïve human populations, underlines the need for the development of safe and effective vaccines. There are safe and effective vaccines that are in human clinical trials including the YF-17D, JEV-17D, and live-attenuated TBEV vaccines. For these vaccines, the most robust correlates of protection are high-titer neutralizing antibodies that protect against severe disease [174,175]. Other flaviviruses such as ZKV and WNV lack safe and effective vaccines or have more limited market potential. Other vaccines are complicated by serotype heterogeneity like DENV, where vaccines must elicit protection against four antigenically distinct serotypes, as well as multiple genotypic variants within a serotype [164,176,177]. In support of neutralizing antibodies as correlates of protection, DENV4 vaccine recipients demonstrated high neutralizing titers and protection from naturally circulating genotype II DENV4 strains while lower neutralizing titers and breakthrough infection were more frequently associated with DENV4 genotype I infection [177,178,179]. Other potential correlates of protection include high T cell responses, high NS1 antibody titers, and, potentially, FcR-mediated effector functions [180,181,182].

A major challenge for developing a safe and effective DENV vaccine involves formulating a balanced yet broadly protective immunity against all four serotypes, simultaneously. Thus, DENV vaccines differ significantly from YF, JEV, and TBEV monovalent vaccines. A major concern in the development of a DENV vaccine is the potential to increase the DENV pathogenesis by inducing low levels of non-neutralizing antibodies to one or more serotypes after tetravalent vaccination, which after DENV infection increases the risk for DSS/DHF [183]. Another consideration is the possibility of eliciting antibody responses that have the capacity to enhance other flavivirus infections, like ZIKV. A recent study showed that sera from DENV-infected individuals elicited long-term antibody responses that recognize E proteins on recombinant ZIKV and either neutralize or enhance infection in vitro in a concentration-dependent manner and in pregnancy models [184,185,186]. However, it remains uncertain as to whether antibodies can enhance ZIKV disease severity in humans [187].

The DENV E protein is the primary target of the neutralizing antibody response in the context of natural infections and is a logical target for vaccine design. In the DENV3 E protein, EDIII mediates receptor binding and cell fusion and is an important target for the humoral immune response [188,189]. EDIII has the highest variability within the serotypes, which makes it an attractive target for generating serotype-specific neutralizing antibody responses that may be less likely to mediate ADE across heterologous serotypes [188]. Because of its role in viral entry and its type-specific antibody response, EDIII has historically been regarded as an ideal antigen in vaccine design [188]. More recently, human monoclonal and polyclonal antibody mapping studies have revealed that natural primary infections typically elicit broad neutralizing antibody responses targeting EDII over EDI and EDIII. In contrast, vaccines may preferentially elicit an EDIII response, suggesting that raising type-specific antibodies to EDII or a combination of two or more ED-specific epitope regions may be critical correlates associated with robust protection from reinfection [186,190,191].

Dengvaxia (Sanofi Pasteur, CYD-TDV) was the first licensed tetravalent DENV vaccine for human clinical use. More recently, QDENGA^®^ (Dengue Tetravalent Vaccine [Live, Attenuated]) (TAK-003), a second live-attenuated tetravalent DENV vaccine, has been approved for use in several countries, offering hope for control of this serious human pathogen. Dengvaxia was developed by using the YF-17D virus as a backbone and engineered to encode the DENV1–4 prM and E sequences as a YF/DENV chimeric live-attenuated vaccine [192]. The vaccine, administered as a three-dose regimen following a 6-month interval between vaccinations, was tested in two phase 3 clinical trials and one phase 2b trial among DENV endemic regions in Latin America and Asian-Pacific countries [193]. Among the 35,000 children that participated (ages 2–16) in the clinical trials, efficacy rates for symptomatic DENV 25 months after a third dose was 60.3% overall, 65.6% in children older than 9 years of age, and 44.6% for children younger than 9 years of age [193]. Additionally, the vaccine’s protection against hospitalization was 72.6% among all participants, 80.75% for children above 9 years of age, and 55.8% for children younger than 9 years of age [193]. In dengue-seronegative children, however, Dengvaxia vaccine recipients were at higher hospitalization risk after natural DENV exposure [194]. In another study, a single dose of CYD-TDV protected in preimmune, but did not protect against dengue infection in naive children [195]. Because of the increased risk of hospitalization and severe DENV observed in individuals younger than 9 years old, Dengvaxia is contraindicated in children under 9 years of age [196].

There are no FDA-approved vaccines for ZIKV to date. However, several preclinical vaccine concepts are in development, including live-attenuated vaccines, inactivated whole virus vaccines, envelope subunit vaccines, messenger RNA (mRNA) vaccines that encode prM and E, DNA vaccines, protein vaccines, and vector-based vaccines [196]. Current issues involving ZIKV vaccine development are the lack of well-characterized animal models, the influence of pre-existing flavivirus infections, the theoretical risk of inducing Guillain–Barré syndrome with live-attenuated virus platforms, as well as safety concerns involving vaccination of pregnant women with live-attenuated vaccines [197]. Attenuated live virus vaccines almost certainly will be contraindicated for pregnant females, as an appropriate vaccine formulation must elicit robust protection in the mother that also protects against in utero ZIKV infection and the onset of microcephaly in the developing infant, who experience the most serious disease outcomes after infection. Sadly, the impact of vaccine-induced or natural infection-induced inflammation during first trimester pregnancies on the developing fetal brain is not fully understood [198].

VRC5288 and VRC5283 are two DNA ZIKV vaccine candidates that have completed phase 1 clinical trials. VRC5288 encodes E proteins from both ZIKV and JEV, while VRC5283 uses an E protein from wild-type ZIKV. Both vaccine candidates elicited elevated levels of neutralizing antibody responses in non-human primates with two doses and reduced viral loads with one dose. The vaccine candidates were then advanced to phase 1 clinical trials [199]. In human volunteers, vaccination with VRC5288 produced antibody responses ranging from 60–89% four weeks after the final boost. Comparatively, vaccination with VRC5283 achieved 100% neutralization via a three-dose needle-free syringe administration. Due to the superior neutralizing antibody response, VRC5283 has advanced into phase II clinical trials [200]. Despite recent advances in delivery and design, DNA vaccines oftentimes elicit reduced neutralizing antibody titers as compared to other vaccine modalities and the potential for DNA vector insertion into the human genome remains an important safety concern [201]. A ZIKV mRNA-LNP vaccine has also proven effective in rodents and primates, providing a robust alternative for human use [202].

Twenty years following the WNV epidemic that occurred in the United States, there is still no approved WNV vaccine licensed for human use, although there are four vaccines available for veterinary use. Three of the veterinary vaccines are based on whole-inactivated viruses and the fourth is based on WNV prM/E expressed in a canarypox-based vector (ALVAC) backbone [203,204]. An important aspect of generating protective immunity against different flaviviruses is the requirement of generating robust immunity in different human populations including the elderly, which do not always efficiently respond to vaccinations and sometimes require higher vaccine doses [205]. A challenge in generating live virus vaccines, which are attenuated, is the reduced growth kinetics in mosquitos in the event of a host–vector transmission event. Transmission of the live virus vaccine into a mosquito population could select for unforeseen mutations in the vaccine strain, leading to reversion to virulence and transmission from arthropod vectors. While human–mosquito transmission is possible, other flavivirus vaccines such as JEV SA14-14-2 and YF-17D showed reduced competence in mosquitos, with JEV Sa14-14-2 being unable to be amplified in pigs from infection of inoculated mosquitos [206,207].

Several WNV vaccines have been tested in human clinical trials including: Chimerivax-WNV02 and rWN/DEN4Δ30, two live-attenuated vaccines [208,209,210]; HydroVax-001, a hydrogen peroxide-inactivated whole-virion vaccine [211]; and VRC-WNVDNA020-00-VP, a DNA vaccine encoding WNV prM/E [212]. HydroVax-001 was evaluated in a small phase 1 trial. The vaccine was well-tolerated with no major adverse side effects. However, this vaccine candidate was not immunogenic as measured by plaque reduction neutralization test (PRNT_50_) at the lowest dose of 1 mcg, but reached 41% seroconversion based on ELISA [211]. The 4 mcg high dose was more immunogenic, with 31% to 50% seroconversion by PRNT_50_ or complement-enhanced PRNT_50_, respectively. Seroconversion by ELISA reached 75% [211]. The most promising vaccine candidate is ChimeriVax-WNV02 which has been evaluated in phase II clinical trials. ChimeriVax-WNV02 was able to achieve >90% seroconversion by PRNT_50_ in healthy adults above the age of 50, 28 days after vaccination. It showed high immunogenicity and was well tolerated among the testing individuals [209]. Previously, a chimeric vaccine PreveNile (Intervet) for horses was approved in 2006, but was removed in 2010 for causing adverse effects such as acute anaphylaxis, colic, and respiratory distress [213].

Several recent reviews that highlight and discuss existing live-attenuated tetravalent vaccines against DENV and other specific flaviviruses vaccines are available in the literature [214,215,216,217].

### New Advances in Flavivirus E glycoprotein Vaccine Design

**Figure 3:**
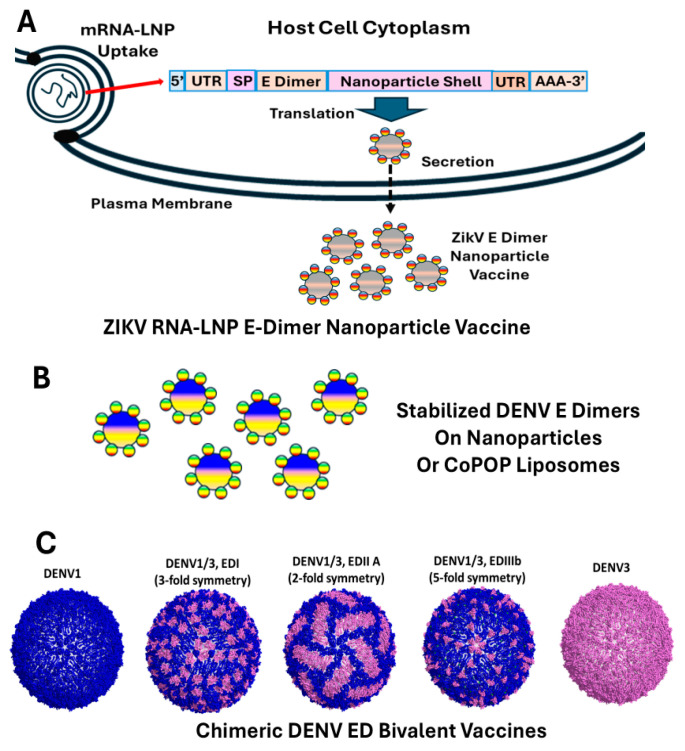
New vaccine designs. (**A**) Lipid nanoparticle delivery of mRNA-encoding nanoparticle shells with surface-expressed Zika envelope dimer proteins. (**B**) Stabilized dengue E dimers expressed on the surface of nanoparticle or CoPoP lipid vehicles [218]. (**C**) Representation of chimeric dengue envelope domain-bivalent vaccine; dengue 1 envelope residues represented in blue, and dengue 3 residues represented in pink [190].

New advances in protein design and the use of mRNA-LNP vaccine formulations have the potential to revolutionize flavivirus vaccine design and performance, while potentially eliminating vaccine-associated complications (Figure 3A–C). Major neutralizing epitopes are scattered across EDI, EDII, and EDIII of the DENV E glycoprotein from different serotypes and type-specific neutralizing antibody responses are proposed as an important DENV vaccine correlate [164]. One new strategy to enhance the breadth of type-specific neutralizing antibody responses after infection or vaccination centers around the design of chimeric immunogens encoding admixtures of EDI, EDII, and EDIII from two or more DENV serotypes [164,190,191]. Recombinant viruses encoding different ED combinations from two or more DENV serotypes oftentimes evolve stabilizing mutations that occur between monomers and at dimer–dimer interfaces (Figure 3C). These recombinant chimeric viruses not only replicate efficiently but elicit bivalent type-specific neutralizing antibody responses in primates, providing new immunogen designs for incorporation into stabilized subunit vaccines for delivery on nanoparticles or via mRNA-LNP [123].

Subunit vaccines provide novel opportunities for vaccine design; however, the existence of potent quaternary epitopes in flaviviruses like DENV and other virus strains is not appropriately presented, diminishing the power of this technology [36,219,220,221,222] and limiting in vivo performance [223,224]. However, recent developments in virus protein design, coupled with stabilizing mutations and new delivery platforms, have revolutionized the availability of new robust strategies for flavivirus vaccine design. These include discoveries in protein structure and function that have promoted prefusion viral proteins, as recombinant protein vaccines and as antigens delivered on nanoparticles or mRNA-LNP platforms (Figure 3A,B) [225,226,227]. A second major advance in protein immunogen design is the identification and application of stabilizing mutations (e.g., interprotomer disulfide bonds, pi–cation and electrostatic interactions, proline substitutions, etc.), which enhance the presentation of authentic virus quaternary epitopes presented in virion encoded glycoproteins and virus-like particle vaccines for protective immunity [221,228,229]. For DENV E glycoproteins, interprotomer disulfide bonds have been used to prevent the presentation of fusion loop-enhancing epitopes, while retaining efficient presentation of the E dimer epitopes, which elicit broadly cross-neutralizing DENV1–4 antibodies [229,230]. Another important feature in these protein designs is the introduction of mutations in the fusion loop, which attenuate the production of weak neutralizing/strong enhancing antibodies that might elicit ADE phenotypes [231]. By introducing mutations within the fusion loop region of DENV, enhancing antibodies can also be subverted and potentially incorporated into recombinant protein vaccine platforms [231]. A second strategy of DENV E dimer stabilization used pi–cation and electrostatic interactions to stabilize DENV dimers, preserving critical type-specific and broadly cross-reactive quaternary epitopes in these vaccine recombinant proteins [228]. Mice immunized with stabilized dimers, but not monomers, developed high levels of DENV2-neutralizing antibodies that bind quaternary epitopes and protect mice immunized with WT E antigen. Consequently, these approaches, alone or coupled with the design of stabilized chimeric ED protein recombinant proteins, offer robust strategies for eliciting antibodies across complex quaternary epitopes encoded within flavivirus virions and can be delivered as adjuvanted recombinant proteins or in nanoparticle or mRNA-LNP vaccines.

Nanoparticle vaccines present chemically cross-linked immunogens on an inorganic (e.g., ferritin, gold, etc.) or organic carrier, typically in lipid carriers, and have been reviewed in the recent literature [232,233]. More recently, structural genes from ZIKV have been expressed from lipid nanoparticles mRNA, which have elicited robust neutralizing antibody responses that protected against lethal infection in AG129 mice [234]. Thus, new technologies offer the emergence of combinatory vaccine platforms including nanoparticle designed flavivirus immunogens, presented from mRNA-LNP vaccine formulations. In parallel, stabilized flavivirus dimers and trimers should easily be incorporated into mRNA-LNP platforms (Figure 2B), offering opportunities to elicit robust quaternary neutralizing antibody responses against different flaviviruses. Although neither of these strategies have been published to date, both offer novel opportunities for vaccine design and delivery.

## 5. Summary

Flaviviruses are positive-sense, single-stranded RNA arthropod-borne viruses that are endemic in the tropical and subtropical regions of the world. The virus family has a long history of causing epidemic and pandemic disease outbreaks in humans, causing considerable disease morbidity and mortality. While the disease burden is high, especially in subtropical and tropical regions, the geographic range of the key vectors is rapidly expanding. Assuming sufficient global support for basic and applied research, recent developments in vaccine and immunogen design offer considerable hope for reducing the disease burden globally. Therapeutic treatments such as monoclonal antibodies offer an effective strategy at treating viral infections by targeting key epitopes on surface proteins preventing virus entry and are generally well-accepted by the body. Although therapeutic approaches to these viruses are limited with no virus-specific FDA-approved treatment available for most flaviviruses, and hampered by difficulties in differential diagnosis, new first-in-class DENV small-molecule inhibitors that are currently in clinical testing offer considerable hope for future generations across the globe. These first-in-class drugs also demonstrate the feasibility for drug discovery to develop additional broadly potent anti-flavivirus drugs. Safe and effective vaccines are available for yellow fever, Japanese encephalitis, and tick-borne encephalitis. But challenges remain, particularly for dengue and the ability to protect against all four serotypes without inducing enhancement. New advancements in mRNA-LNP vaccine design offer the potential to generate effective vaccines while eliminating complications. Potential strategies to increase antibody response include creating chimeric immunogens to envelope protein subtypes, recombinant viruses that contain different envelope domain configurations from different dengue serotypes, and preventing the generation of weak neutralizing antibodies. Animal models, specifically mice, remain a critical liability in the development of therapeutic drugs and vaccines against flaviviruses, and represent an important future research direction for the field. Animal models in immunocompetent hosts remain a critical research gap. Overall, flavivirus research appears well-poised to discover and deliver new drugs, therapeutics, and vaccines for improved global health in the 21st century.
